# Design and validation of a high-speed hyperspectral laparoscopic imaging system

**DOI:** 10.1117/1.JBO.29.9.093506

**Published:** 2024-08-13

**Authors:** Kelden Pruitt, Ling Ma, Armand Rathgeb, Jeffrey C. Gahan, Brett A. Johnson, Douglas W. Strand, Baowei Fei

**Affiliations:** a University of Texas at Dallas, Center for Imaging and Surgical Innovation, Richardson, Texas, United States; b University of Texas at Dallas, Department of Bioengineering, Richardson, Texas, United States; c University of Texas Southwestern Medical Center, Department of Urology, Dallas, Texas, United States; d University of Texas Southwestern Medical Center, Department of Radiology, Dallas, Texas, United States

**Keywords:** hyperspectral imaging, minimally invasive surgery, laparoscopy, high-speed, image processing, system validation

## Abstract

**Significance:**

Minimally invasive surgery (MIS) has shown vast improvement over open surgery by reducing post-operative stays, intraoperative blood loss, and infection rates. However, in spite of these improvements, there are still prevalent issues surrounding MIS that may be addressed through hyperspectral imaging (HSI). We present a laparoscopic HSI system to further advance the field of MIS.

**Aim:**

We present an imaging system that integrates high-speed HSI technology with a clinical laparoscopic setup and validate the system’s accuracy and functionality. Different configurations that cover the visible (VIS) to near-infrared (NIR) range of electromagnetism are assessed by gauging the spectral fidelity and spatial resolution of each hyperspectral camera.

**Approach:**

Standard Spectralon reflectance tiles were used to provide ground truth spectral footprints to compare with those acquired by our system using the root mean squared error (RMSE). Demosaicing techniques were investigated and used to measure and improve spatial resolution, which was assessed with a USAF resolution test target. A perception-based image quality evaluator was used to assess the demosaicing techniques we developed. Two configurations of the system were developed for evaluation. The functionality of the system was investigated in a phantom study and by imaging *ex vivo* tissues.

**Results:**

Multiple configurations of our system were tested, each covering different spectral ranges, including VIS (460 to 600 nm), red/NIR (RNIR) (610 to 850 nm), and NIR (665 to 950 nm). Each configuration is capable of achieving real-time imaging speeds of up to 20 frames per second. RMSE values of 3.51±2.03%, 3.43±0.84%, and 3.47% were achieved for the VIS, RNIR, and NIR systems, respectively. We obtained sub-millimeter resolution using our demosaicing techniques.

**Conclusions:**

We developed and validated a high-speed hyperspectral laparoscopic imaging system. The HSI system can be used as an intraoperative imaging tool for tissue classification during laparoscopic surgery.

## 1 Introduction

Laparoscopic and robotic surgery has been increasing in prevalence across all surgical specialties for decades, in fields that include urology,^1^ neurology,^2^ and gynecology.^3^ This has resulted in shorter hospital stays, decreased intraoperative blood loss, and decreased infection rates in patients.^4^^,^^5^ As minimally invasive surgery (MIS) and techniques have evolved and improved, surgical robotics and novel imaging systems have further advanced the field and enhanced surgeon capabilities in various procedures. This includes the development of fluorescence imaging (FI) systems,^6^ 3D stereoscopic visualization technology,^7^ and narrow-band imaging (NBI) tools,^8^ all of which have been incorporated into MIS imaging systems with the hope of reducing adverse outcomes such as anastomotic leakage occurrence rates,^9^ iatrogenic injuries,^10^ and positive tumor margins.^11^ As these technologies have been shown to improve surgical outcomes, they have seen increased translation in modern, clinical systems. As such, we will briefly cover each in more depth.

Stereoscopic visualization enables 3D visualization of the operative field by transmitting two video feeds into each eye of the surgeon, mimicking depth perception in the human brain.^12^ The benefits of this technology are largely subjective, with comparative studies showing a general preference among surgeons toward 3D visualization.^13^ Commercial systems such as the da Vinci Robot (Intuitive Surgical Inc., Sunnyvale, California, United States) have incorporated this technology in their current surgical platforms. FI, on the other hand, enables enhanced visualization of anatomical features including vasculature,^14^^,^^15^ sentinel lymph nodes,^16^^,^^17^ and tumors.^18^^,^^19^ Indocyanine green (ICG) is the most commonly used fluorophore, as it is Food and Drug Administration–approved (along with 5-aminolevulinic acid for glioma imaging), but others have been explored for potential applications, including methylene blue and IRDye®. Many clinical systems include FI modalities with ICG, including the da Vinci Firefly mode and the PINPOINT imaging system from Stryker. NBI is an optical imaging modality that filters out red light, focusing on blue and green light using narrow optical filters.^8^ This imaging technique has been popularized by Olympus imaging systems for neoplasia detection^20^ and endometriosis identification,^21^ among other applications. These state-of-the-art systems still have drawbacks such as the quick half-life and saturation effect of ICG or the lack of objective, quantitative analysis in real time.^22^^,^^23^ Although studies using these fluorescent agents have shown promising results, more work needs to be done toward achieving quantifiable results and solidifying patient benefits.^24^ Likewise, the contribution of NBI to MIS is subjective as well, with the technology serving as a visualization aide rather than as a diagnostic tool.

Hyperspectral imaging (HSI) has been investigated for non-invasive, label-free, and quantitative applications as it captures spectral and spatial information without the need for contrast agents.^25^ HSI has been used to perform semantic segmentation tasks, distinguishing different tissue types when used alongside various classification techniques. Seidlitz et al.^26^ demonstrated the superiority of HSI in performing semantic segmentation across 19 classes, outperforming both normal RGB data and tissue parameter images—including tissue oxygenation, perfusion, water, and hemoglobin indices displayed as heatmaps of the original image. That said, the ability to produce tissue parameter images and measure blood oxygen saturation through HSI^27^ in intestinal tissues remains useful in other applications—such as colorectal anastomoses.^28^^,^^29^ Jansen-Winkeln et al.^29^ found that HSI effectively assessed perfusion and aided in determining an optimal resection line for successful anastomosis. HSI is also used in disease diagnosis, histopathology, and surgical guidance.^25^^,^^30^ Although the promise of HSI and its contributions are apparent, it is not without drawbacks. Current systems are often bulky, disallowing translation into MIS.^29^^,^^31^ Acquisition times have improved but can remain on the order of seconds,^32^ which is unsuitable for MIS, where motion blur and other artifacts may affect image quality.^33^ Further development and better system design are needed to translate HSI into a real-time operative workflow.

Our work seeks to address these drawbacks by developing a high-speed HSI setup for laparoscopic applications through the use of spectrally resolved detector arrays (SRDAs). SRDAs are capable of real-time spectral imaging thanks to the micro-scale optical filters overlaid on their complementary metal-oxide semiconductor imaging sensors. This technology has been used for retinal imaging where minimal light exposure is necessary^34^ and has been used in flexible endoscopy for esophageal applications.^35^ Although this technology allows fast image acquisition, it generally comes at the cost of reduced spectral and/or spatial information as fewer wavelengths with smaller raster sizes are captured than in their push-broom or spectral scanning counterparts.^36^^,^^37^ To address these drawbacks, we propose a system that leverages the imaging speed capabilities of snapshot hyperspectral imagers combined with demosaicing techniques for improved spatial resolution and a dual-camera (DC) configuration to mitigate the relatively fewer wavelengths captured by the snapshot cameras individually.^38^^,^^39^ Luthman et al.^35^ implemented an imaging setup with two SRDAs for esophageal applications using a flexible Polyscope. We present a more compact, lightweight, and mobile setup for hyperspectral dual-camera rigid laparoscopy. Overall, high-speed HSI configurations, e.g. single-camera (SC) and DC, have yet to be fully developed, integrated, and applied in a single system. We propose to advance this technology by constructing and validating a laparoscopic imaging system and exploring potential applications.

## 2 Methods

Our HSI system is easily integrated into typical intracorporeal laparoscopic setups with the imaging system being fixed to the eyepiece of a clinical laparoscope. This section outlines the equipment, techniques, and procedures we underwent to construct and test our laparoscopic HSI system.

### 2.1 Imaging Setup

**Fig. 1:**
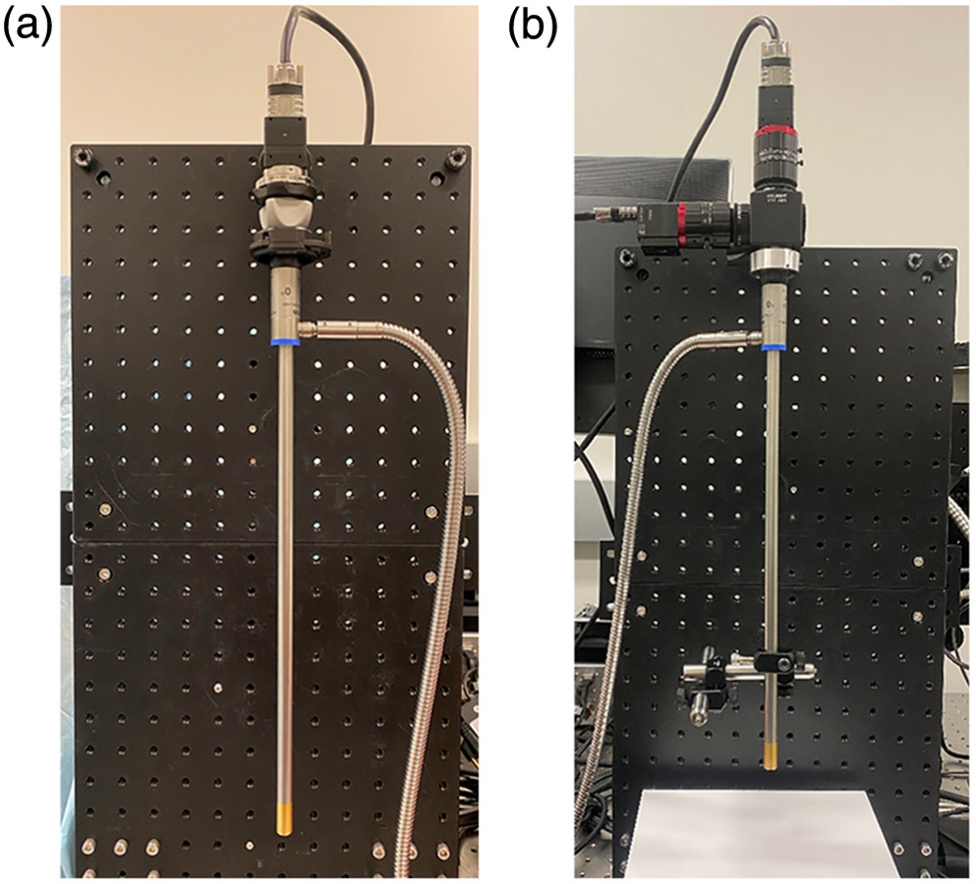
Laparoscopic HSI system configurations. (a) Single camera. (b) Dual camera.

The imaging setup included a 10-mm, 0-deg laparoscope (WA4KL100 UHD, Olympus America, Center Valley, Pennsylvania, United States) and a 150-W halogen source (OSL2IR High-Intensity Fiber-Coupled Illuminator, Thorlabs, Newton, New Jersey, United States) with an operating wavelength range of 400 to 1750 nm. The SC configuration in Fig. 1(a) includes a 20-mm C-mount adapter (Stryker, Kalamazoo, Michigan, United States) and a 2× C-mount fixed focal length lens extender. In this work, three snapshot hyperspectral cameras with different wavelength profiles were used. The visible (VIS) hyperspectral camera utilizes a 4×4 mosaic pattern and captures 16 bands covering a spectral range of 460 to 600 nm. Meanwhile, the red/near-infrared (RNIR) camera covers 15 wavelength bands ranging from 600 to 870 nm with a 4×4 mosaic configuration as well. Finally, the near-infrared (NIR) hyperspectral camera utilizes a 5×5 mosaic configuration and captures 24 bands from 660 to 960 nm. Each camera weighs 32 g, leading to a total additional weight to the SC setup of 162 g with the attached optics (not including the laparoscope itself). This setup was used for all spectral and spatial evaluations, as well as the phantom study. For the *ex vivo* studies, the DC configuration shown in Fig. 1(b) was utilized, which includes a 605-nm dichroic mirror (Thorlabs), two 35-mm VIS-NIR C-mount lenses (Edmund Optics, Barrington, New Jersey, United States), and two snapshot hyperspectral cameras capable of capturing up to 120  hypercubes/s. Fabricated adapters couple the eyepiece of the laparoscope to the dichroic mirror, and another adapter was made to attach the fiber light guide from the halogen source to the light post of the scope.

### 2.2 Image Acquisition

Once the imaging system was constructed, various considerations were taken for controlled, accurate, reproducible image acquisition. First, all imaging was done at a working distance of 7 cm. An adjustable lab jack (Thorlabs) was used to ensure a fixed working distance for each procedure. We then focused on the respective hyperspectral cameras and set the exposure time to allow maximal intensity when imaging a white reference (WR) reflectance tile without saturating the imaging sensor. Other optical parameters of the system were set as well, including f/#, focal length, and exit pupil distance. Last, before acquiring images of the target/tissue, we acquired white and dark reference images for HSI calibration. The dark reference/dark current image must be acquired by either shutting off the light source or blocking any incoming light at the distal end of the scope. Conversely, the WR image was taken with a 95% reflectance surface (Spectralon, Labsphere Inc., North Sutton, New Hampshire, United States) at the approximate working distance of the target or specimen. To mitigate the effects of noise, we employed temporal averaging by capturing multiple hyperspectral images of both the tile and the imaged target/tissue. For each validation procedure, ∼30 images were acquired for each tile and target. We increased this for the *ex vivo* study, imaging each sample ∼65 times. Room lights were turned off to avoid variation due to stray light during acquisition.

### 2.3 Software Development

**Fig. 2:**
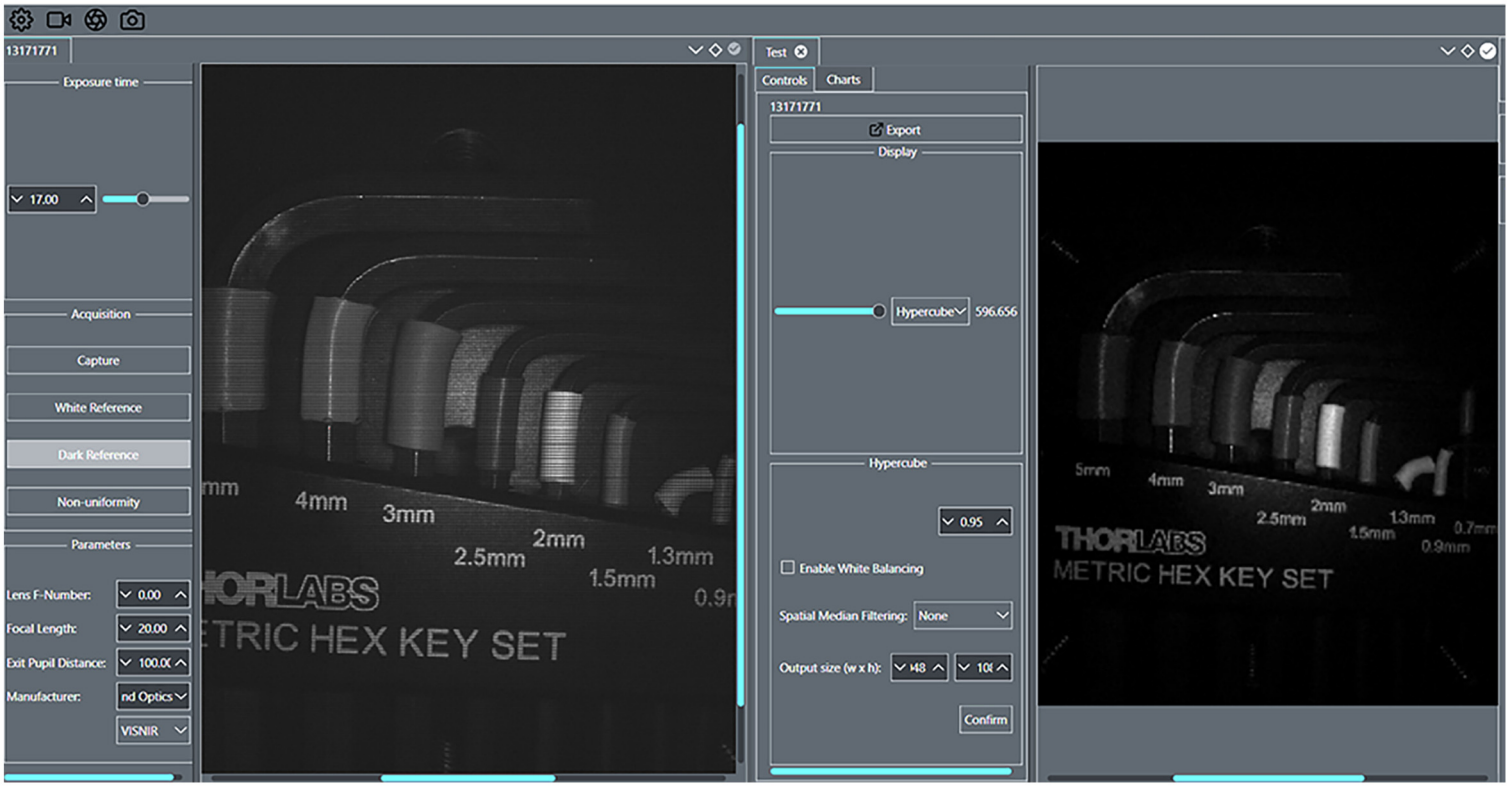
Software application for concurrent hyperspectral imaging with the dual-camera system. The left pane shows the live video feed of the camera as raw, mosaic images. The right pane shows a single band from a captured hypercube.

To efficiently acquire and process images with the system, we developed an in-house software application written in C++ using the QT graphical user interface framework. This enables the consolidation of image acquisition, processing, and implementation of tools and features, such as demosaicing and temporal averaging, in a single interface. The application, as a universal tool, allows up to five hyperspectral cameras to be connected simultaneously with the runtime parameters for acquisition and processing individually set for each camera. This allows the simultaneous acquisition of hyperspectral images with multiple cameras, a feature leveraged in our DC configuration. For efficient imaging with our stationary system, a burst capture feature was implemented, allowing users to specify a number of images or an amount of time for which to capture. Figure 2 shows an example of hypercube acquisition using a snapshot camera.

### 2.4 System Validation

To validate the spectral accuracy of the hyperspectral cameras in the system, standard reflectance tiles were used (Spectralon, Labsphere Inc.) to compare the spectral footprints from hyperspectral images with standard reflectance curves generated with a calibrated spectrophotometer. The used tiles are characteristically Lambertian in their reflectance profile, unlike the tissues used in our *ex vivo* study. Eight tiles with different colors were included in the spectral analysis. Each tile produces a unique spectral footprint throughout the wavelength range of 360 to 830 nm, allowing us to validate the accuracy throughout the intensity range of the system. Another reflectance tile with a calibrated spectral profile extending into the NIR range was used to assess the NIR hyperspectral camera (WCS-MC-020, Labsphere Inc.). The spectral accuracy of the system was quantitatively evaluated using the root mean squared error (RMSE) metric. To evaluate the spatial resolution capabilities of the setup, a 1951 USAF Resolution Test Target (Thorlabs) was imaged, and Michelson contrast was calculated. To assess the system’s capabilities when imaging non-Lambertian surfaces such as those seen intraoperatively, spectral validation with *ex vivo* tissues was performed with a calibrated spectrometer (HR2000+ES, Ocean Insight, Orlando, Florida, United States) by fixing the probe in the field of view (FOV) of the laparoscope so that ground truth spectral footprints of the tissue in the hyperspectral camera FOV could be measured for comparison. The spectrometer was calibrated using a radiometrically calibrated source (HL-3P-CAL, Ocean Insight) for absolute irradiance measurements from 190 to 1100 nm.

### 2.5 Demosaicing

**Fig. 3:**
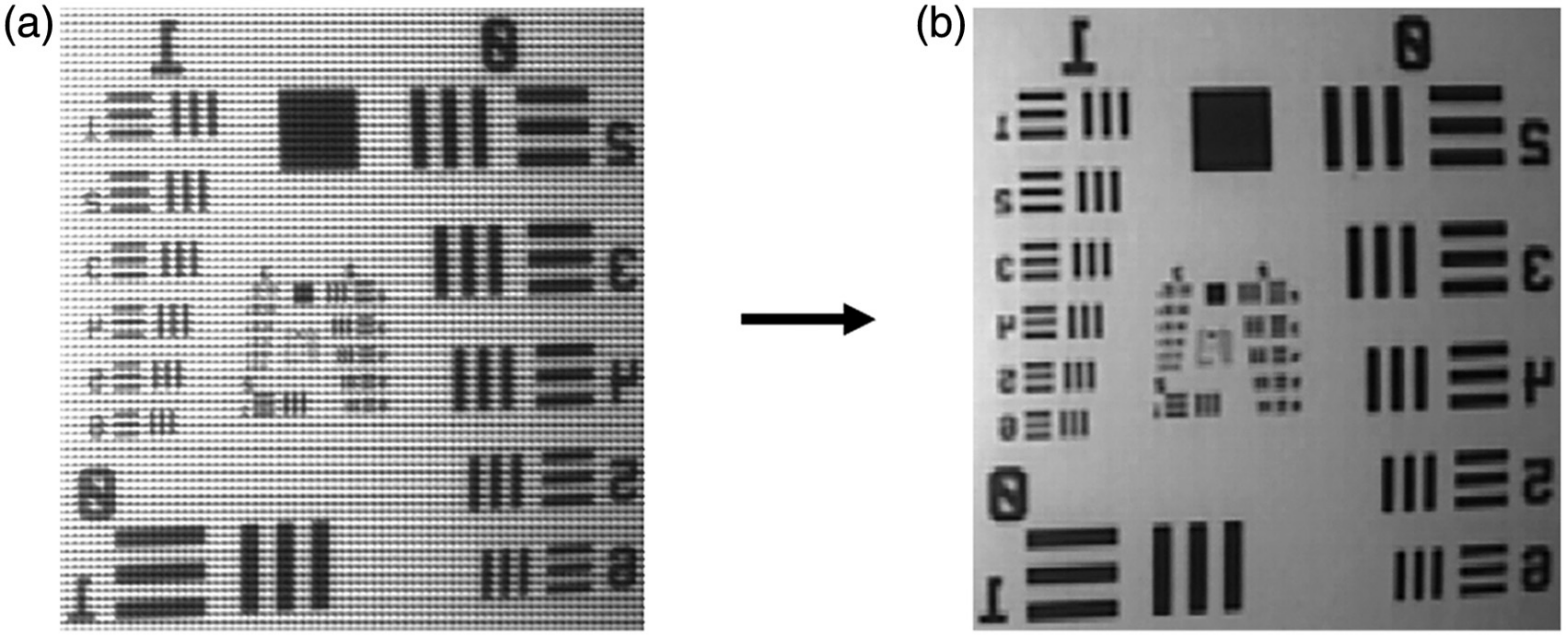
Conceptual visualization of demosaicing. (a) Raw mosaic image is processed into the (b) demosaiced image.

Unprocessed images acquired with SRDAs produce an image with higher resolution than the final processed hypercube, as the entire image sensor is used. However, due to the repeated pattern of micro-scale optical filters and different relative transmissions, a “mosaic” pattern overlays the grayscale image. To take advantage of the larger image raster size of the unprocessed images and their potential to improve the spatial resolution of the system, we performed a demosaicing process on the unprocessed, mosaic images. This process can be visualized in Fig. 3, which shows the removal of the mosaic pattern seen throughout the image. Three demosaicing methods were evaluated for their performance: white reference calibration (WRC), low-pass filtering (LPF), and filter convolution (FC).

The WRC technique is straightforward—deriving its name from the common HSI preprocessing step. This method requires the mosaic image of a WR tile in conjunction with the image to be demosaiced (referred to as the original image henceforth). The final image is produced via element-wise division of the original image by the WR mosaic

$$I_{\text{demosaiced}}(x,y)=\frac{I_{\text{raw}}(x,y)}{I_{\mathrm{WR}}(x,y)},$$ (1)

where $I_{\text{demosaiced}}$ is the final demosaiced image intensity, $I_{\text{raw}}$ is the intensity value of the raw image that needs to be demosaiced, and $I_{\mathrm{WR}}$ is the intensity value of the WR mosaic, all at index (x,y).

The LPF method was developed by intuiting that the repeated mosaic pattern can be treated as a form of high-frequency noise that can be filtered out using standard image processing techniques. Therefore, the pattern may be removed by performing a 2D Fourier transform on the original image, isolating the magnitude component, and then applying a threshold to remove high-frequency signals from the magnitude plot. We made sure to retain those signals that occur in the central region of the plot, as it includes low-frequency information vital to the image. Finally, an inverse 2D Fourier transform was performed to recover the newly demosaiced image. The threshold value was determined experimentally for the test target data.

**Fig. 4:**
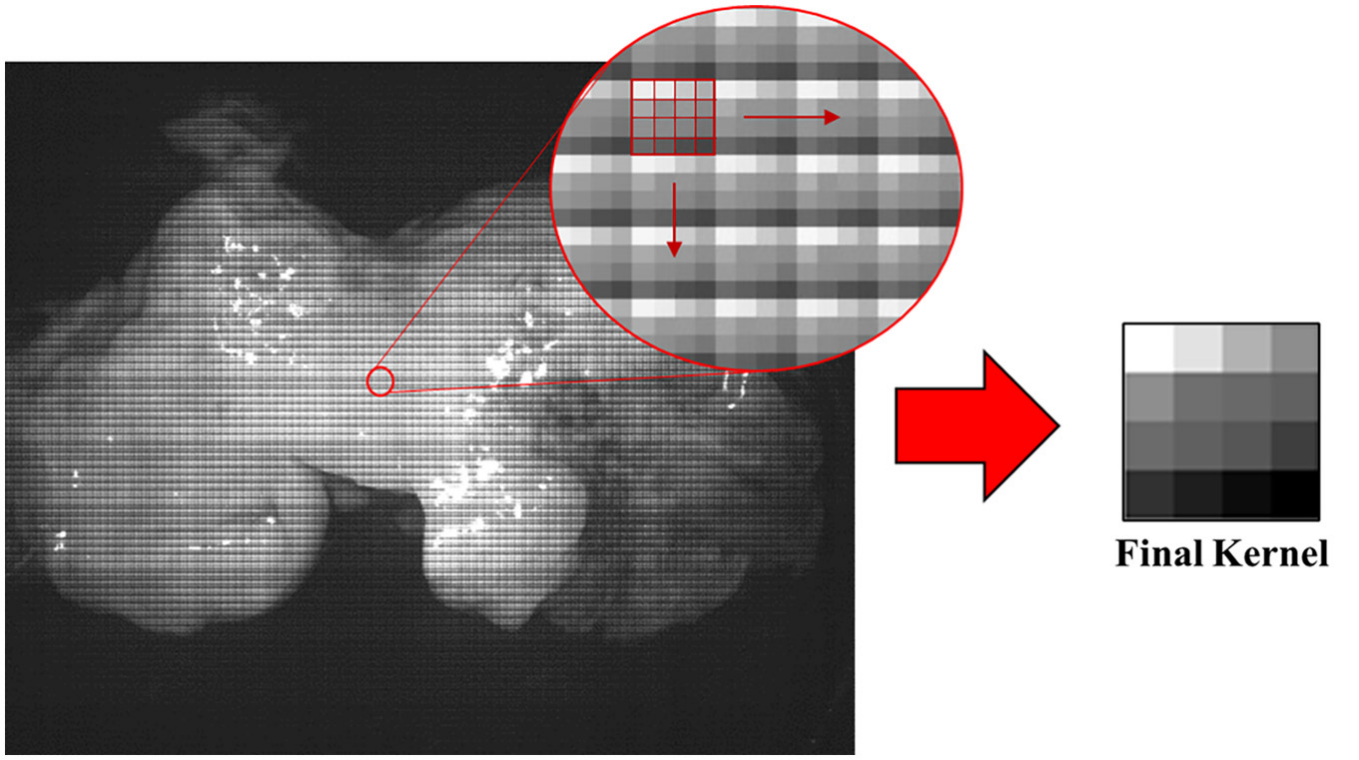
Kernel formation. The final kernel is the resultant matrix of a sum performed across the original image at each filter sub-unit. The final filter characterizes the response of each filter in the array.

**Fig. 5:**
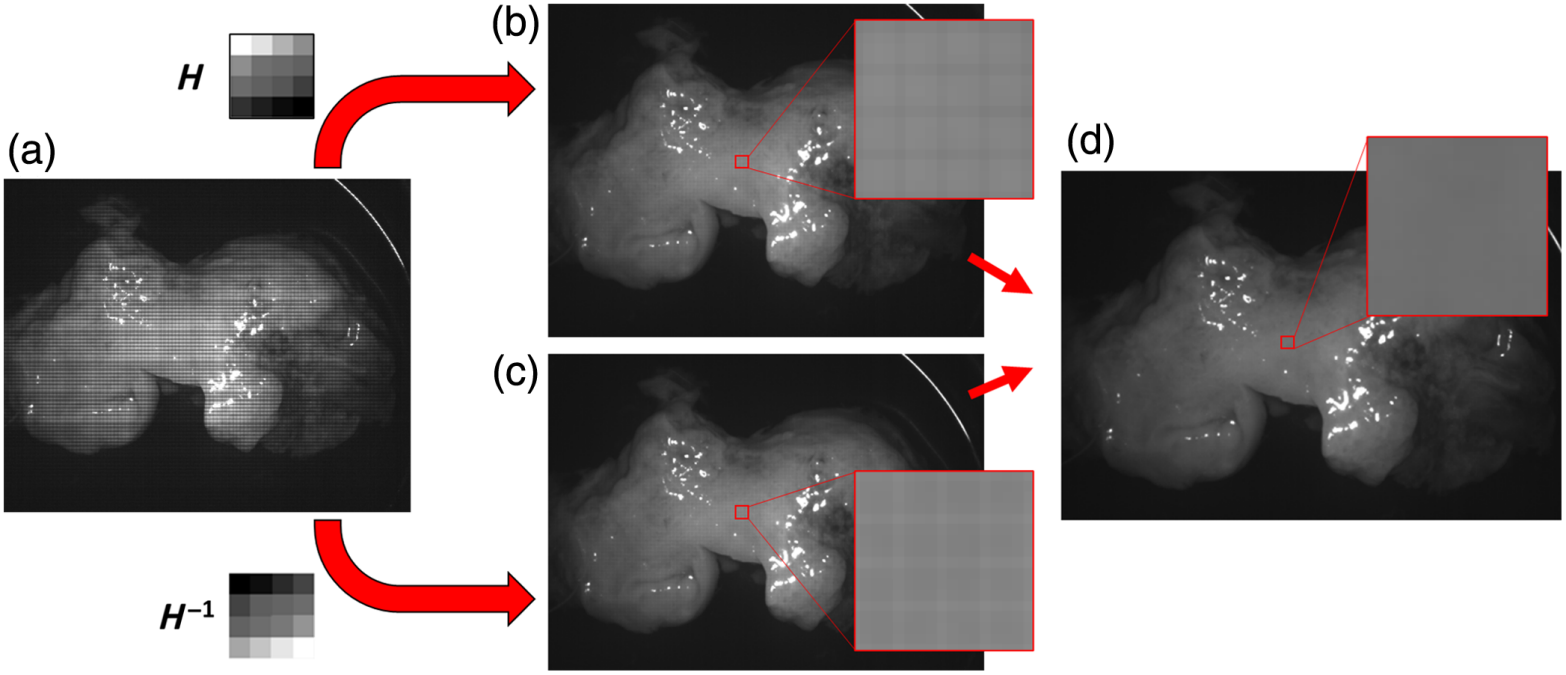
Filter convolution demosaic method. (a) Original mosaic image. (b) Resulting image from convolution performed with filter H. (c) Resulting image from convolution performed with filter $H^{-1}$. (d) Final demosaiced image.

Last, the FC method was assessed. We form two kernels related to the filter responses of the hyperspectral camera. The first was formed by performing a filter-wise sum across the original image to produce a kernel equivalent in size to the mosaic configuration of the camera, either 4×4 or 5×5. Figure 4 shows the kernel formation process. The second kernel is the reciprocal of the first. Each kernel is convolved separately over the original image. The two resulting images were normalized and averaged to form the final demosaiced image. Finally, an unsharp filter is applied to increase image sharpness.^40^ An overview of the FC method is shown in Fig. 5.

### 2.6 Spatial Resolution Evaluation and Quality Evaluation

To measure spatial contrast, a Michelson contrast cutoff of 20% was used as shown in Eq. (2)

$$\text{Contrast}=\frac{S_{\max}-S_{\min}}{S_{\max}+S_{\min}}.$$ (2)

**Fig. 6:**
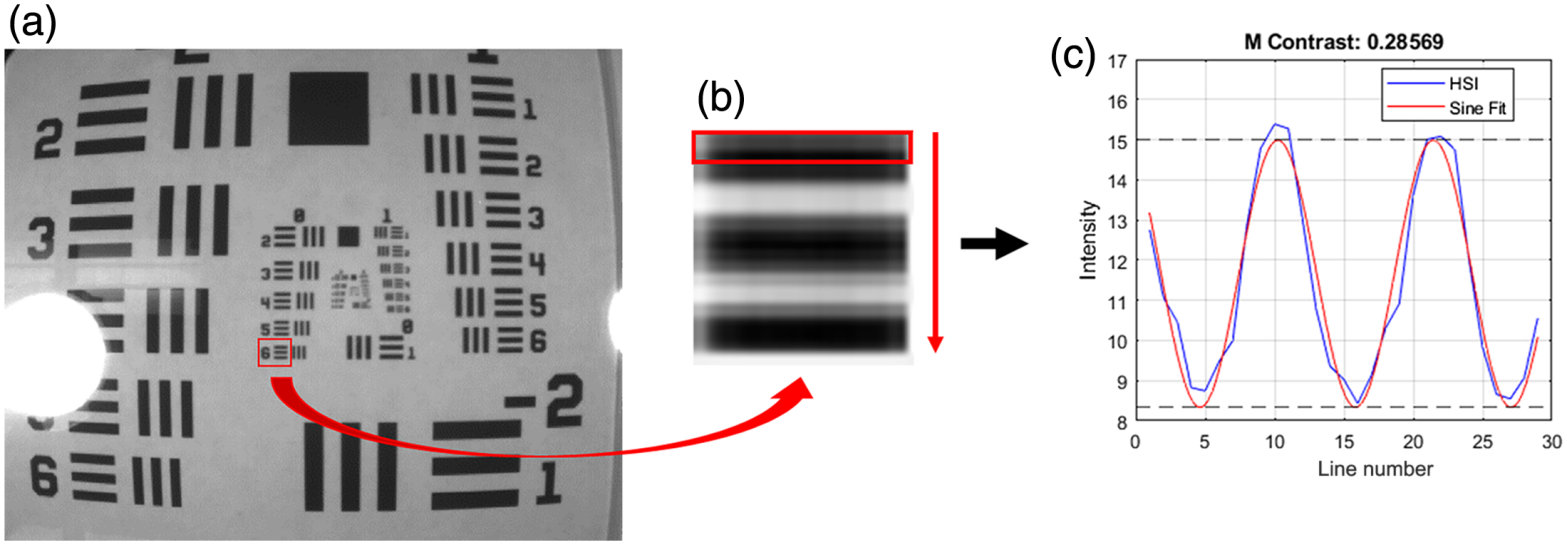
Spatial resolution assessment. (a) Demosaiced image of the test target. (b) Line elements being analyzed. (c) Resulting sinusoidal curve from the group of line elements.

As shown in Fig. 6, the line elements were analyzed to produce a plot of intensities along the line pairs. A sine curve was then fit to the plot to get $S_{\max}$ and $S_{\min}$ values. $S_{\max}$ corresponds to the peak of the sine curve produced by the intensities of the line elements, and $S_{\min}$ corresponds to the minimum of the curve. Smaller elements and groups of the resolution target were analyzed until the contrast threshold was surpassed. The contrast was analyzed on full-resolution demosaiced images, including WRC, LPF, and FC methods outlined in Sec. 2.5.

In addition to gauging the resolution using contrast, we evaluated our demosaicing techniques using a perception-based image quality evaluator (PIQUE).^41^ PIQUE is an unsupervised and opinion-unaware no-reference algorithm that estimates distortion and local variance, with lower scores indicating higher-quality images. As image brightness affects PIQUE scores, the demosaiced images were normalized and brought to similar brightness levels by scaling each image to the same mean intensity over the assessed region to produce comparable results.

### 2.7 *Ex Vivo* Tissue Imaging

**Fig. 7:**
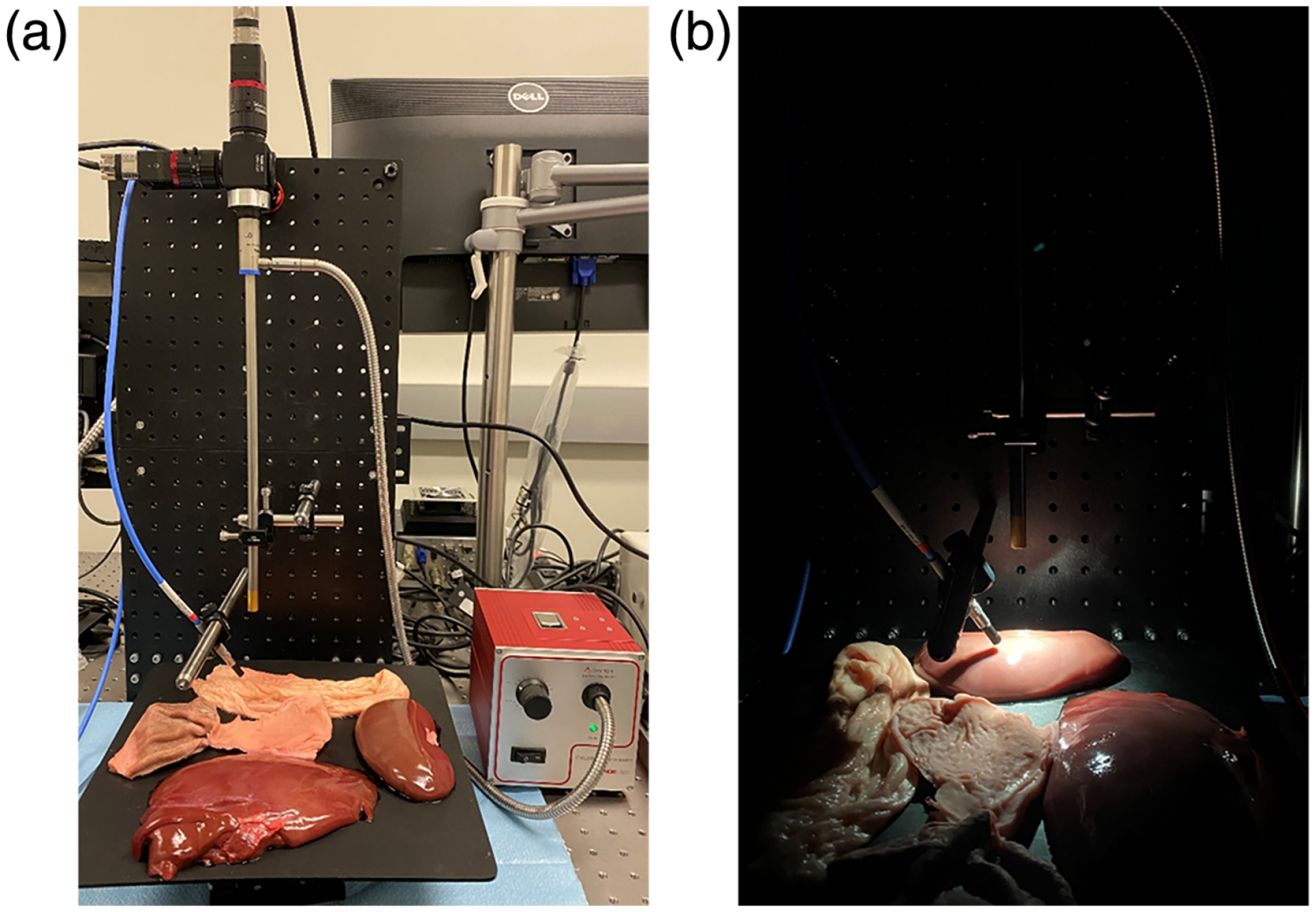
Imaging system and spectrometer placement over *ex vivo* tissue scene. (a) Full imaging system view. (b) Active imaging view.

To further validate our system for laparoscopic imaging, porcine visceral organs were used for *ex vivo* tissue imaging, including the stomach, liver, intestine, and kidney. To cover a larger spectral range and avoid the need to change cameras, our DC configuration was used with the VIS and RNIR hyperspectral cameras. Specimens were imaged at a real-time rate of 25 frames per second for 50 to 60 unprocessed frames. After hypercube reconstruction, temporal averaging was performed to reduce the effect of noise on the hyperspectral images and subsequently generated spectral curves. Ground truth spectral reflectance values were obtained via a radiometrically calibrated spectrometer alongside our system to validate the spectra of the tissues. Absolute reflectance values from the tissues were then linearly transformed for comparison with the relative reflectance produced by the system. Figure 7 shows the imaging setup used for imaging the visceral tissues and capturing simultaneous ground truth spectral curves from the spectrometer. Manual segmentation of the hyperspectral images from each camera was performed to produce masks for registration among the cameras. Preprocessing for registration included averaging the hypercube along the spectral dimension to produce a single grayscale image and binarizing each resulting image at an experimentally determined threshold. Further refinement was performed before performing affine, control point registration of the RNIR hypercube to the VIS hypercube. As the cameras were fixed within the system, the same transformation matrix could be used for each hyperspectral image. A proposed region of interest (ROI) corresponding with the captured spectrometer readings was assumed for each tissue for comparison of spectral curves.

**Fig. 8:**
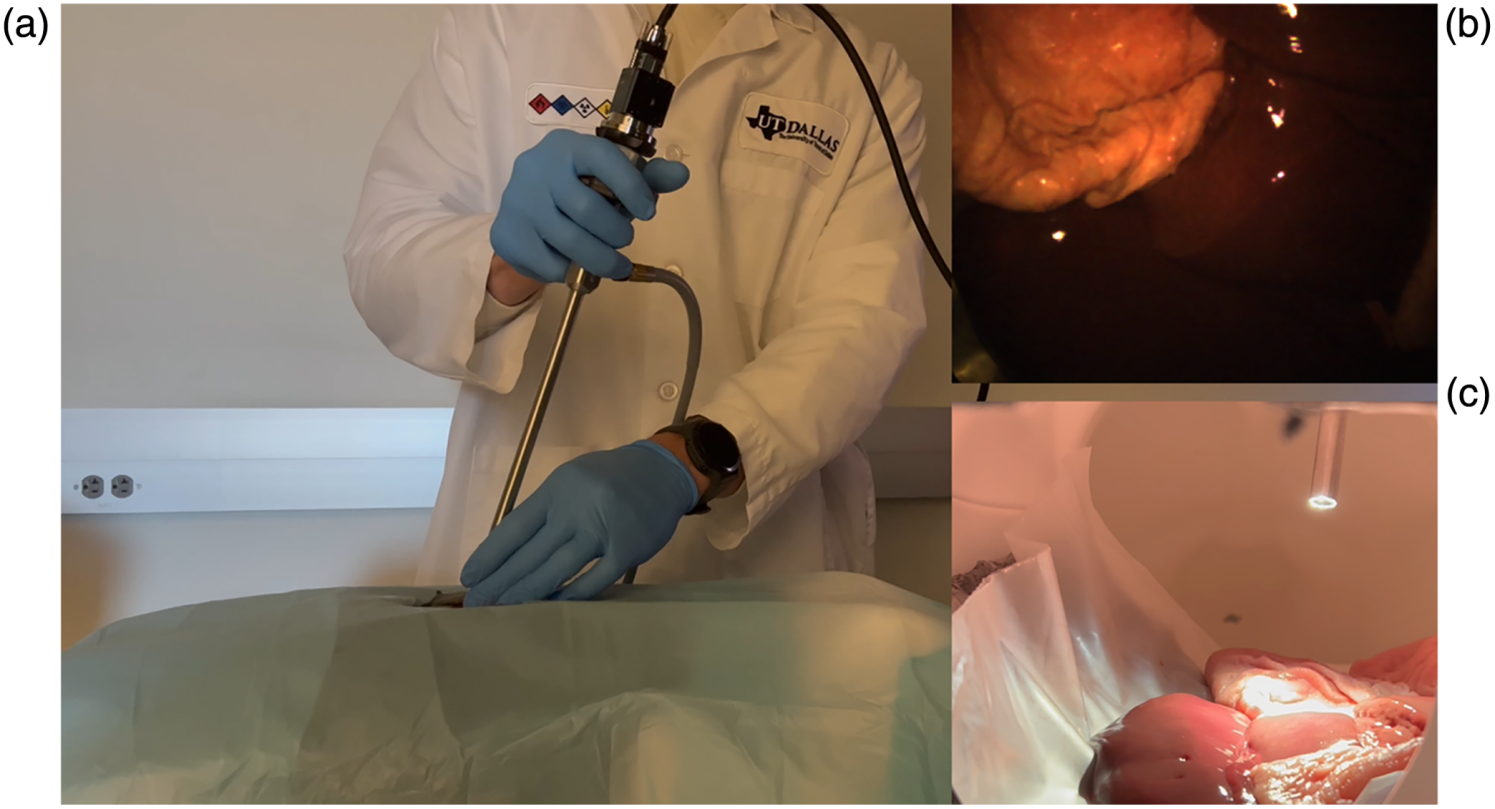
Phantom assessment of laparoscopic HSI system. (a) External view of SC configuration. (b) Pseudo-RGB formed from the hyperspectral frame in a video sequence. (c) Internal view of the distal end of the laparoscope and visceral tissues.

To further simulate intracorporeal procedures, abdominal tissues were positioned within a phantom model, and a hyperspectral image sequence was captured, as depicted in Fig. 8. In this procedure, a porcine liver, intestine, and two kidneys were introduced into the phantom for imaging. This facilitated the examination of the system’s mobility and maneuverability in a dynamic environment, which is more akin to abdominal MIS than the static setting used for controlled imaging and experimentation. The phantom consisted of a modified hollow mannequin portion to accommodate *ex vivo* tissues. We bored a 0.5-in. (1.27-cm) aperture in the abdomen below the umbilicus for the insertion of the 10-mm laparoscope.

### 2.8 Tissue Spectra and Pseudo-RGB Construction

To analyze the tissue spectra derived from the hyperspectral cameras, a binary mask of the tissue was generated. Hyperspectral images were calibrated using white and dark reference images according to our previous work.^38^ Pseudo-RGB images were created for each tissue image by selecting the nearest bands from the VIS hyperspectral camera corresponding to RGB color channels, 700.0, 546.1, and 435.8 nm, respectively. Calibrated reflectance values were then acquired over ROIs approximating the FOV of the spectrometer as gauged by pseudo-RGB images to produce the spectral footprints of each imaged tissue. A threshold was applied to the pixels in the ROI to mitigate the effects of glare. Glare pixels were not removed outright as this processing could not be mirrored in the ground truth spectroscopy data.

## 3 Results

### 3.1 Spectral Accuracy and Spatial Resolution

**Fig. 9:**
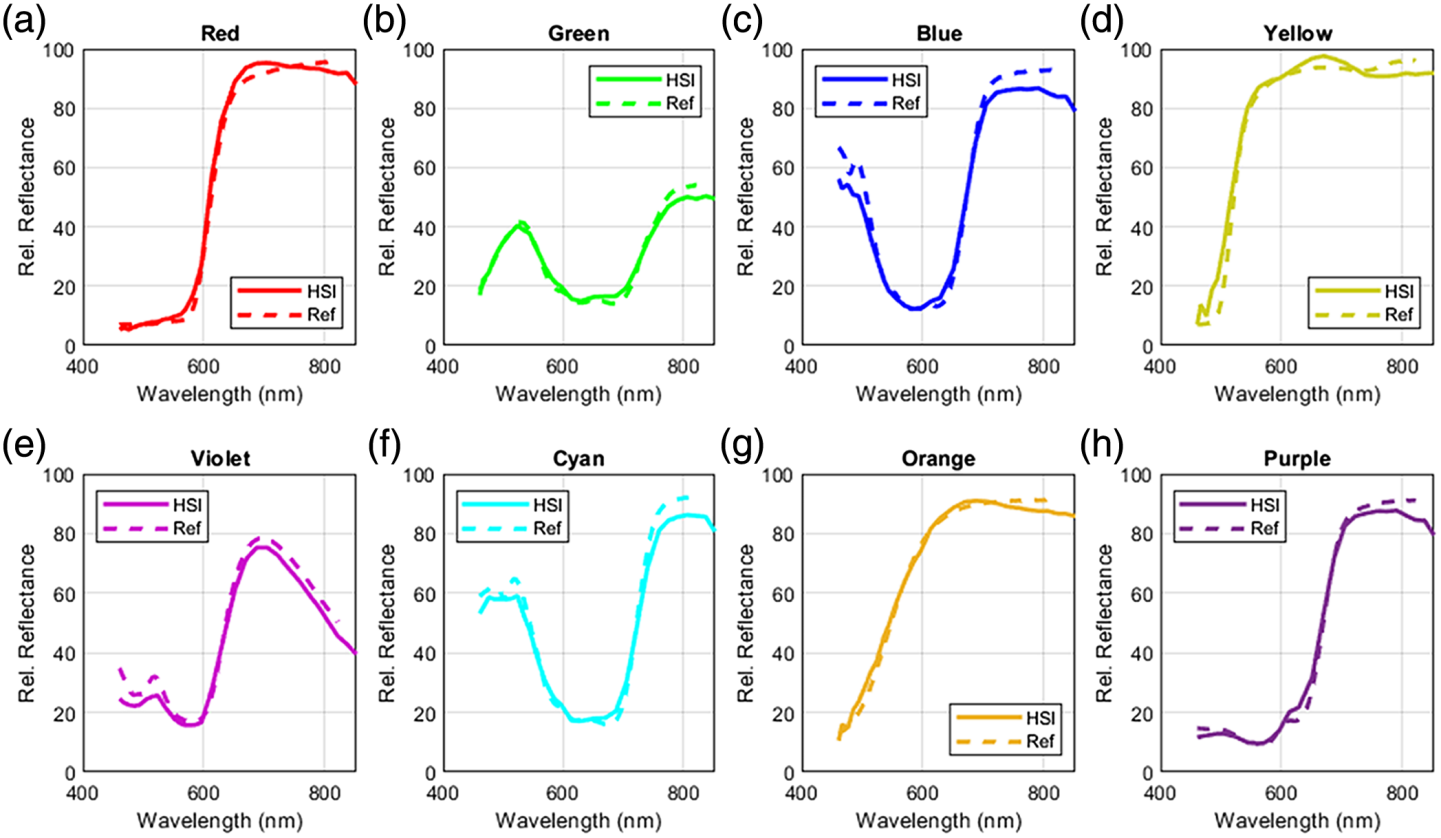
Result of the spectral accuracy of VIS and RNIR cameras. HSI spectral curves (solid) against the tile standard reflectance (dashed). (a) Red reflectance tile. (b) Green reflectance tile. (c) Blue reflectance tile. (d) Yellow reflectance tile. (e) Violet reflectance tile. (f) Cyan reflectance tile. (g) Orange reflectance tile. (h) Purple reflectance tile.

**Fig. 10:**
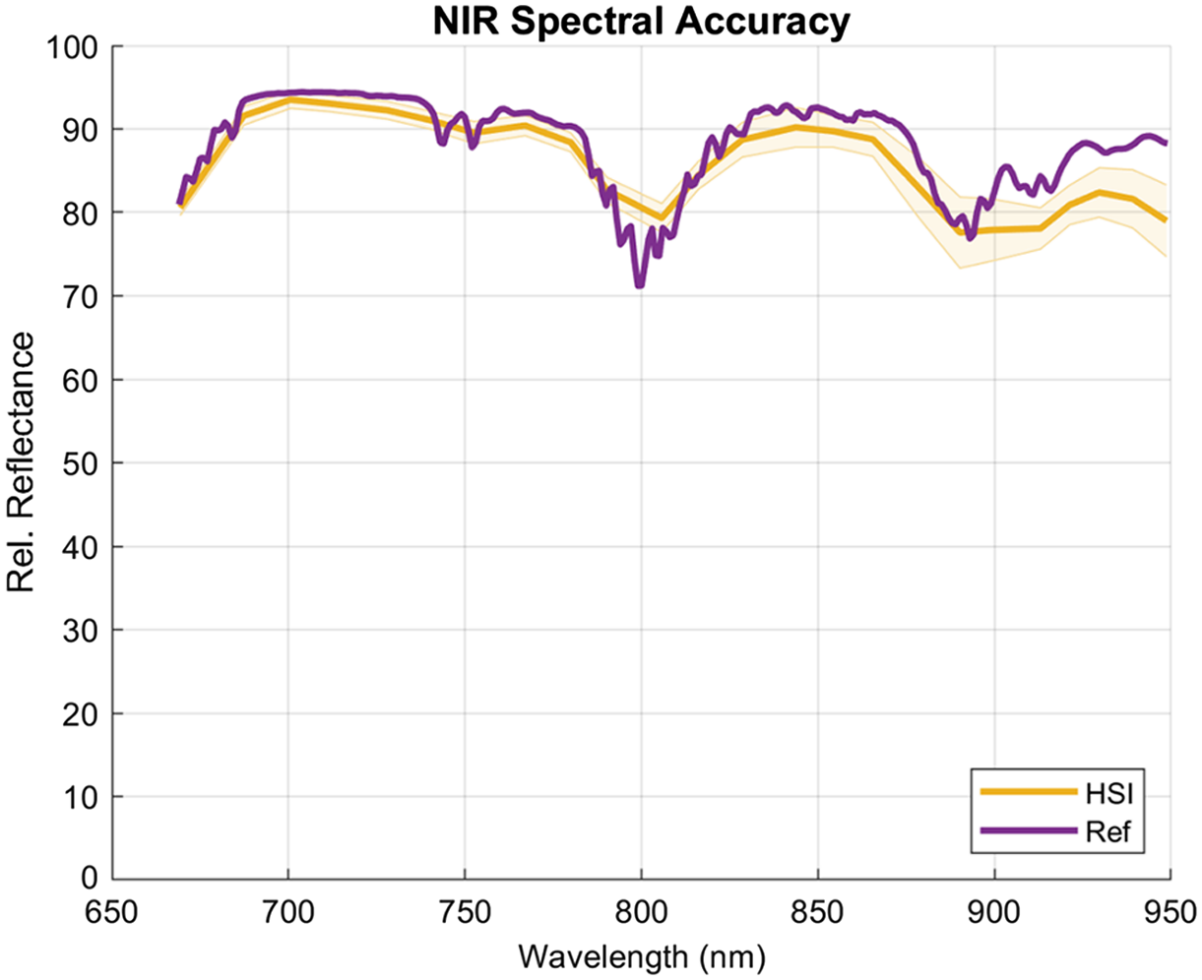
Result of the spectral accuracy assessment for the NIR hyperspectral camera. HSI (yellow) plotted as mean ± SD (shaded area) versus reference curve (purple).

Spectral validation was performed on the reference tiles mentioned in Sec. 2.4 with the SC configuration of each camera. Spectral footprints of each of the eight color tiles were used to assess the VIS and RNIR hyperspectral cameras. The spectra covered a range of 360 to 830 nm, with a 1-nm step size. For spectral validation of the NIR camera, the NIR tile is used with a calibration spectral footprint that covers 235 to 2000 nm, with a 1-nm step size as well. The VIS and RNIR hyperspectral cameras covered 461 to 597 and 614 to 853 nm, respectively, with the VIS capturing 16 wavelengths and the RNIR capturing 15 wavelengths. As a result, the HSI curve of the RNIR camera extended marginally beyond the reference curves for the tiles. These values were excluded from the quantitative analysis. The resulting spectral curves from the VIS and RNIR cameras are plotted in Fig. 9 along with the ground truth calibration curves. The NIR hyperspectral camera captured a spectral range of 669 to 949 nm, and its resulting spectra are shown in Fig. 10. Overall, the recorded reflectance values from each of the systems conformed to the reference curves well with the largest RMSE of the VIS camera (blue = 6.25 and yellow = 6.31) resulting from a slight shift in the steep region of the tile reflection profile. The error also seemed to accumulate in the extreme regions of the captured spectral range, especially toward the longer wavelength region of each system, as seen in the blue spectral curve and RMSE of the RNIR camera and the NIR spectral plot. The standard reflectance values of the tiles used overall cover a broad range of intensities, showing the sensitivity of the system to accurately discern spectral features at different relative reflectance intensities. Overall, the average RMSEs achieved by the VIS and RNIR cameras were 3.51±2.03% and 3.43±0.84%, respectively, whereas the NIR camera achieved an RMSE of 3.47%. Table 1 outlines the RMSE values for each tile.

**Table 1:** RMSE for each reflectance tile.

Cam	Tile									
	Red	Green	Blue	Yellow	Violet	Cyan	Orange	Purple	NIR	Average
VIS	2.01	1.32	6.25	6.31	4.66	3.45	2.68	1.41	—	3.51
RNIR	3.29	2.52	4.94	3.21	3.37	4.27	2.39	3.42	—	3.43
NIR	—	—	—	—	—	—	—	—	3.47	—

**Fig. 11:**
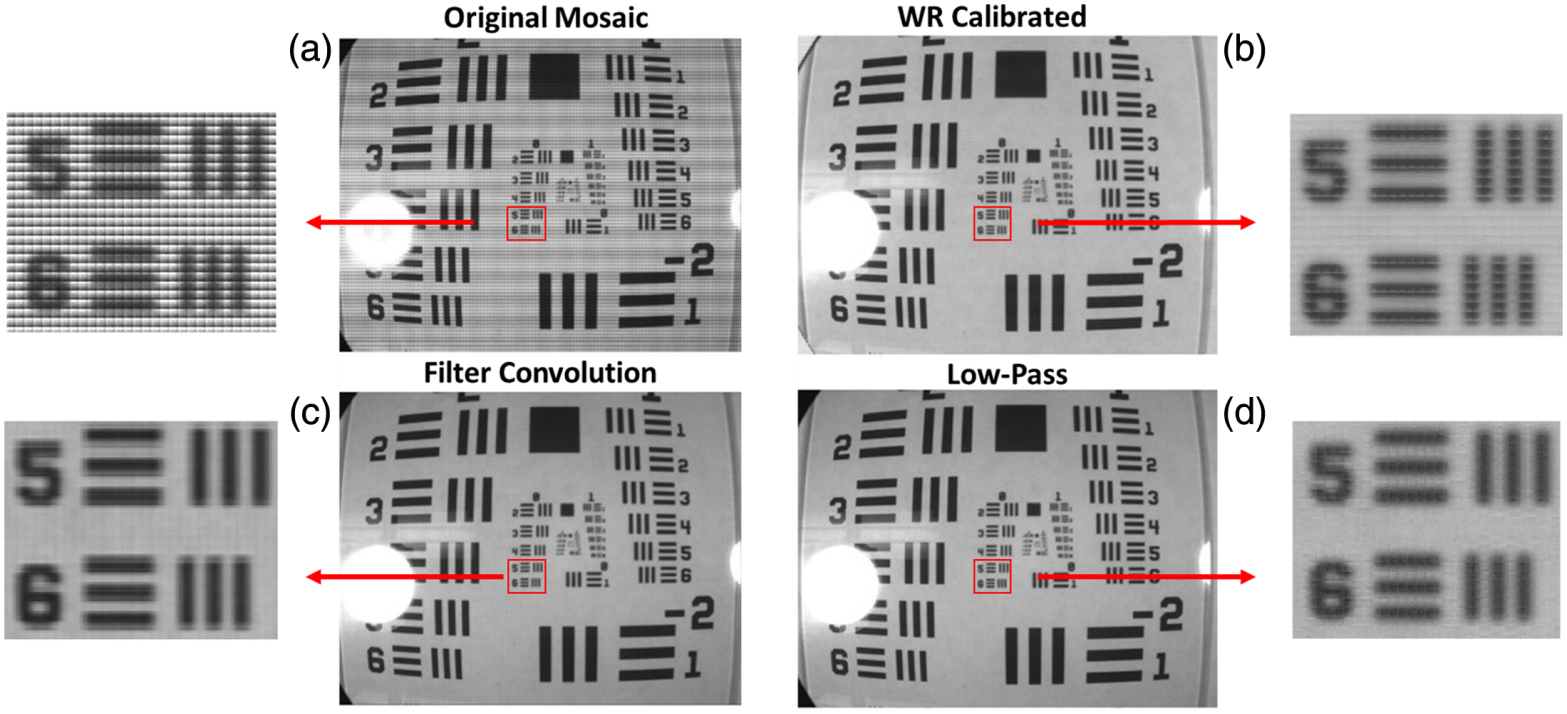
Qualitative results of each demosaicing method. (a) Original mosaic image. (b) WR calibration demosaic output. (c) Filter convolution demosaic output. (d) Low-pass demosaic output.

Spatial validation showed sub-millimeter horizontal and vertical resolution for each camera and each demosaicing technique (besides vertical resolution with NIR camera with WRC) as shown in Table 2. The WRC demosaic narrowly outperformed FC, followed by LPF for the VIS camera. Vertical and horizontal resolutions of 0.250 and 0.223 mm were achieved, respectively. For the RNIR camera, FC was superior, equally distinguishing vertical and horizontal elements spaced 0.353 mm apart. The other two techniques performed equally with the RNIR camera in each direction, achieving 0.500 and 0.446 mm vertical and horizontal resolutions, respectively. Resolutions achieved with the NIR camera were larger than those of the other cameras likely due to the larger mosaic configuration of 5×5. It’s also worth noting that the reflectance profile of the line elements of the test target is progressively more reflective moving into the RNIR–NIR wavelength range, also contributing to decreased contrast and overall performance of the RNIR and NIR cameras. That said, FC outperformed the other techniques by a significant margin for the NIR camera. Besides the performance of the VIS camera, the FC technique is the superior demosaicing method for optimal spatial resolution. Qualitative analysis suggests its superiority as well, as LPF results retain some high-frequency information and WRC outputs seemingly lack global contrast, each resulting in lower-quality images. The qualitative results of each method are shown in Fig. 11 as a small region of each image is highlighted.

**Table 2:** Spatial resolution (mm) and PIQUE scores achieved with each hyperspectral camera.

		WRC	LPF	FC
VIS camera	Vertical	**0.250**	0.397	0.281
	Horizontal	**0.223**	0.353	0.281
	PIQUE	**20.8**	23.7	32.8
RNIR camera	Vertical	0.500	0.500	**0.353**
	Horizontal	0.446	0.446	**0.353**
	PIQUE	35.1	32.8	**26.8**
NIR camera	Vertical	1.000	0.794	**0.500**
	Horizontal	0.893	0.794	**0.500**
	PIQUE	**20.4**	25.4	31.3

Note: bold values indicate the best performing demosaic technique in each metric.

The resulting PIQUE scores from each camera suggest superior quality for the WRC output for both the VIS and NIR cameras despite it being the poorest performing method with the RNIR camera. Instead, the RNIR camera saw superior results from FC demosaicing. There are few global patterns across each camera, suggesting the importance of method selection depending on the given hardware.

### 3.2 *Ex Vivo* Tissue Spectra

**Fig. 12:**
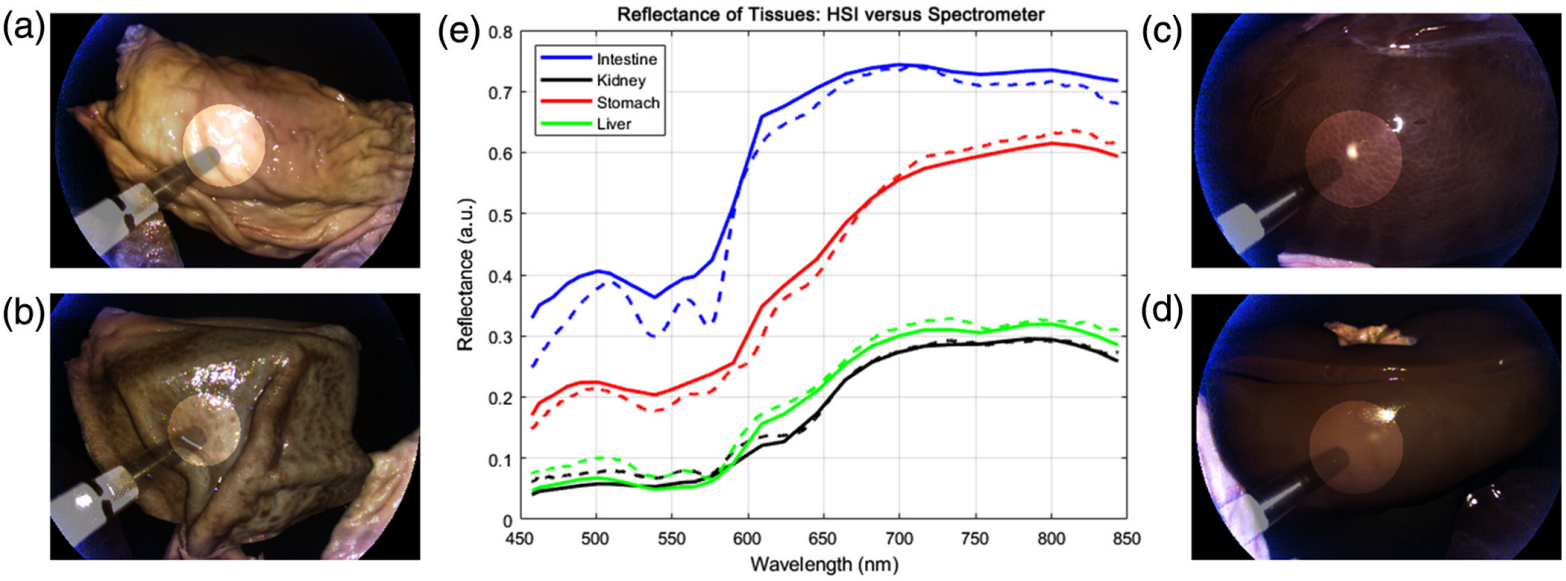
*Ex vivo* experimental results. Pseudo-RGB endoscopic image of (a) intestinal, (b) stomach, (c) liver, and (d) kidney tissues, each with ROI highlighted. (e) Spectral footprint of liver, stomach, intestinal, and kidney tissues from the laparoscopic system (solid) plotted alongside the spectrometer output (dotted).

Tissue imaging was carried out using the DC configuration. Blood absorption coefficients for oxygenated and deoxygenated hemoglobin vary distinctly in the RNIR region, providing clear tissue distinctions and estimation of blood content.^42^ Tissues were imaged individually and collectively, and masks were created for each specimen of spectrometer ROIs after thresholding glare pixels to a maximum value of 1. Figure 12 shows the pseudo-RGB images produced from the VIS hypercubes as well as the mean reflectance values for each tissue within the highlighted ROI throughout the spectral range of the VIS and RNIR cameras. The resulting spectral curves produced from the calibrated spectrometer were also plotted for reference. The values measured by the spectrometer underwent a similar calibration to the hypercubes, as each tissue spectral curve was normalized to the spectral curve of the white reference tile under similar lighting conditions and working distance. After calibration, a least squares regression was performed with one of the tissues to slightly adjust the scale further as the laparoscopic HSI system produces relative reflectance values and not absolute measurements. We note that the specimens are distinguishable by their reflectance intensities, especially in the RNIR range.

As seen in a few of the validation spectral curves of the reference tiles, the features in the short VIS range from the laparoscopic HSI system did not align as well with the spectrometer curves as in other wavelength ranges.

### 3.3 Phantom Imaging

The SC configuration of the system was used to acquire a hyperspectral sequence of images using the VIS camera. The pseudo-RGB feed was visualized for each of the abdominal tissues in the phantom. The system was shown to be maneuverable and lightweight, so as not to disrupt the standard workflow. Significant variation in image brightness was observed with varying working distances without a feature equivalent to auto exposure time as seen in RGB systems.

## 4 Discussion and Conclusion

A high-speed hyperspectral laparoscopic imaging system along with an acquisition and processing framework was developed for MIS applications. The SC setup is shown for validation procedures and a functional phantom study. Our system’s DC configuration approximately doubles the spectral coverage of the system with negligible loss of speed and accuracy. We managed this with improved hardware and software design. This system can be used in future studies to address current setbacks in MIS and improve patient outcomes through objective, quantitative means.

The system was validated to show acceptable spatial resolution and spectral accuracy. Error accumulation was seen at the ends of captured spectral ranges, which is especially seen in the longer wavelength regions of each hyperspectral camera. Our proposed justification for this behavior is the light source used and the transmission of the filters used to form the hypercubes. The halogen source used emits a lower power density of light in the lower visible wavelength range. When tested with a clinical xenon source, this behavior is mitigated. As for the error in the longer wavelength ranges, the filters used for the final RNIR and NIR hyperspectral camera wavelengths along with the underlying sensor technology result in relatively lower quantum efficiencies than other wavelengths captured with the system, likely leading to a decreased signal-to-noise ratio. *Ex vivo* visceral tissues were imaged with the system to mimic tissues visualized during laparoscopic and robotic surgeries and were analyzed according to their respective spectral footprints in both stationary and mobile settings.

The high-speed hyperspectral laparoscopic imaging system can have numerous applications in endoscopic surgery across many specialties. The software tool under development can interface with endoscopic systems and utilize the rich information provided by HSI, as well as provide interpretable results in the future through the development of integrated tools. Our system addresses the drawbacks of long acquisition times and/or bulkiness of current HSI systems, as well as the decreased spectral range of fast systems. Techniques such as temporal averaging and demosaicing are utilized to show sufficient spectral accuracy and spatial resolution in a high-speed, lightweight HSI system for laparoscopic applications. The larger mosaic pattern of the NIR camera and the reflective profile of the material used in the resolution test target are likely contributors to the performance drop seen in the NIR and RNIR cameras when compared with the VIS camera. Future studies and work may be done with regard to varying mosaic sizes and their effect on the spatial resolution achievable by these compact hyperspectral cameras.

The constructed system shows the ability to distinguish different visceral tissues based on their spectral reflectance patterns, a capability that will be built upon in future studies. Kidney and liver tissues are spectrally similar, but there is a notable distinction between the curves produced by our system and the spectrometer as measurements extend beyond the VIS range. That said, tissue drying, glare, field of view, and viewing angle of the spectrometer are all possible confounding variables in the workflow and are considered. Discrepancies seen in the VIS range, particularly highlighted in the acquired spectra from intestinal tissue, are likely due to some of these confounding factors. In particular, alternative glare handling strategies, such as outright removal in the hyperspectral image, may be considered to improve spectral accuracy in some wavelength ranges. Various mitigation strategies can be further investigated. Although the system proved functional in the simulated setting, data validity was difficult to assess and was not thoroughly analyzed. We suspect that the calibration of hyperspectral images will need further consideration as working distances vary intraoperatively, as previous work has shown the inverse square law behavior of light intensity at different working distances.^32^ Future work will also explore the many applications of HSI as it relates to endoscopic, laparoscopic, and robotic surgeries and automated real-time intraoperative histopathological diagnosis, with an emphasis on different spectral ranges for various procedures, with the goal of improving MIS outcomes and patient prognoses.

## Data Availability

Code and data underlying the results presented in this paper are not publicly available at this time but may be obtained from the authors upon reasonable request.
